# Multi-Functional Potential of Lactic Acid Bacteria Strains and Antimicrobial Effects in Minimally Processed Pomegranate (*Punica granatum* L. cv Jolly Red) Arils

**DOI:** 10.3390/microorganisms10101876

**Published:** 2022-09-20

**Authors:** Leila Ben Farhat, Flora Valeria Romeo, Paola Foti, Nunziatina Russo, Cinzia Lucia Randazzo, Cinzia Caggia, Ferid Abidi

**Affiliations:** 1 Laboratory of Protein Engineering and Bioactive Molecules (LR11ES24), INSAT (Institut National des Sciences Appliquées et de Technologie Centre Urbain Nord), University of Carthage, Tunis BP 676-1080, Tunisia; 2 Department of Agriculture, Food and Environment (Di3A), University of Catania, Via S. Sofia 100, 95123 Catania, Italy; 3 CREA (Consiglio per la Ricerca in Agricoltura e l’analisi dell’Economia Agraria), Centro di Ricerca Olivicoltura, Frutticoltura e Agrumicoltura, Corso Savoia 190, 95024 Acireale, Italy; 4 ProBioEtna SRL, Spin-off of University of Catania, Via S. Sofia 100, 95123 Catania, Italy; 5 CERNUT (Interdepartmental Research Centre in Nutraceuticals and Health Products), University of Catania, Via le A. Doria 6, 95125 Catania, Italy

**Keywords:** post-biotics, cell-free supernatant, ready-to-eat fresh fruits, functional foods

## Abstract

This study aimed to evaluate the antimicrobial activity of both cells, and cell-free supernatants (CFS) of 7 selected lactic acid bacteria (LAB) strains belonging to *Limosilactobacillus fermentum* (4 strains), *Lacticaseibacillus paracasei* (1 strain), *Lacticaseibacillus rhamnosus* (1 strain), and *Enterococcus faecium* (1 strain) species, against *Listeria monocytogenes*, *Escherichia coli, Salmonella* Typhimurium, *Pseudomonas aeruginosa* and *Staphylococcus aureus*, by both the agar-well diffusion and co-culture methods. In addition, probiotic and safety traits were also detected. Great variability was detected on antimicrobial effects, whereas all tested strains were found sensitive to most of the tested antibiotics, and without any DNase, gelatinase, or hemolytic activity. Moreover, strains showed excellent survival in acidic conditions and exhibited tolerance to pepsin and bile salts. Based on the in vitro results, the CFSs of two selected *L. fermentum* strains were applied, in a mixed solution, as bio-preservative into minimally processed pomegranate arils, inoculated with a cocktail of *L. monocytogenes* and *E. coli*. Samples, packaged in an ordinary atmosphere, were analyzed during refrigerated storage, for up to 12 days, for physicochemical (as weight loss, texture, color, pH, total soluble solids and organic acid content) and for microbiological traits. Results revealed the effectiveness of CFS, up to 12 days, in reducing weight loss and microbial growth, without any significant effect on texture, total soluble solid content and color, found comparable to the acid citric treatment, highlighting the multi-functional potential of selected probiotic strains.

## 1. Introduction

The global ready-to-eat fresh produce market has rapidly grown in recent years due to changes in consumer food attitudes, and a wide variety of products is available in the market with functional foods positively considered by consumers for their beneficial effects and nutritional traits [1]. Nevertheless, fresh and minimally processed vegetables represent a favorable matrix for both spoilage and pathogenic microorganisms, which cause economic losses and human diseases, such as acute enteritis with fever, bloody diarrhea and pseudo appendicitis [2]. Indeed, ready-to-eat fruits are extremely perishable due to the occurrence of physiological and biochemical changes, with significant degradation of sensorial traits, such as browning and modification of texture and flavor. Several studies reported the presence of pathogenic species, including *Listeria monocytogenes*, *Salmonella enterica*, *Escherichia coli*, *Yersinia enterocolitica*, *Aeromonas hydrophila* and *Staphylococcus aureus* both on fresh produce and minimally processed products [3,4,5,6]. In a recent survey, upon 10,070 samples of fresh fruit and vegetables collected in retail stores, the most isolated microorganisms were *E. coli*, *Salmonella enterica* and *E. coli* O157: H7 [7]. In the last 30 years, different strategies, mainly based on the addition of chemical substances (such as sodium lactate, sodium diacetate, trisodium phosphate, sodium hypochlorite, peroxyacetic acid and salicylic acid) have been applied to avoid both spoilage and foodborne pathogen growth [6,8]. Alternative strategies, based on physical treatments, such as electrolyzed water, high-intensity ultrasound, UV light, pulsed light, ionizing radiation, cold gas plasma and the use of phages have been developed [9,10]. However, none of these strategies is effective in avoiding microbial growth, being unable to completely eradicate microbial presence [11,12].

Pomegranate (*Punica granatum* L.) is a fruit typically growing in the Northern and Southern hemispheres, from September to February, and from March to May. Pomegranate fruits are sources of bioactive compounds with high nutritional value and health-promoting effects [13,14]. Although pomegranates are usually sold as whole fruit, recently the consumer’s demand for ready-to-eat pomegranate arils has increased. The main hurdle for the marketing of the arils is the short shelf life which is not always consistent with the needs of food retailers [15,16]. Moreover, consumers require healthier food without chemical additives and industries continuously propose novel bio-additive compounds. The exploitation of natural bio-preservatives, or addition of lactic acid bacteria (LAB) and/or their metabolites with antagonistic effects against the deleterious microorganisms are continuously proposed [17,18], and successfully applied for controlling the growth of other microorganisms and/or for enhancing the nutritional values [19]. In this scenario, the proposal of applying post-biotics has emerged with the advantage of a lower interaction with the food matrix and greater ease of use [20,21,22]. Postbiotics are defined as the preparation of inanimate microorganisms and/or their components that confers a health benefit on the host [23,24]. Post-biotics include any compounds released or produced through the metabolic activity of microorganisms, such as exopolysaccharides, enzymes, cell wall fragments, short chain fatty acids (SCFAs) or bacterial lysates. The major benefits of post-biotics are their inherent stability in industrial processes and storage, and intellectual property protection (as no live microorganisms can be isolated from post-biotic). Several studies have succeeded in identifying LAB metabolites which mainly include organic acids (acetic, formic, and propionic, among others), fatty acids, hydrogen peroxide (H_2_O_2_), proteinaceous compounds, as bacteriocins (nisin, reutericyclin, pediocin, lacticin, reuterin and sakacin), and some volatiles such as diacetyl, and/or ethyl alcohol [25,26,27]. However, the amount of the metabolites has generally been found in concentrations much lower than their minimal inhibitory concentrations (MICs), indicating that the antimicrobial metabolites might act in synergy [28].

The aim of the present study was to evaluate the antibacterial effects, safety and functional traits of a panel of LAB strains. Moreover, for the first time, the cell-free supernatant (CFS) of the two most promising strains were sprayed, as a mixed solution, on minimally processed pomegranate arils to evaluate its effects on a mixed culture of *L. monocytogenes* and *E. coli*, during refrigerated storage up to 12 days.

## 2. Materials and Methods

### 2.1. Bacterial Strains and Culture Conditions

The LAB used in the present study included strains previously isolated from traditional fermented dairy-products collected in Beja, Ben Arous, Zaghouan and Tunis [29]. LAB strains were genetically identified by 16S rRNA gene sequencing as four strains of *Limosilactobacillus fermentum* LBF4, LBF5, LBF15 and LBF17 (strains accession number OM165034, OM165035, OM165036, OM165038, respectively), *Lacticaseibacillus paracasei* LBF19 (strain accession number OM171261), *Lacticaseibacillus rhamnosus* LBF16 (strain accession number OM165037), and *Enterococcus faecium* LBF20 (strain accession number OM165039). All strains belong to the Dairy Science Department, Faculty of Agriculture, Kafr El-Sheikh University, Egypt.

LAB strains were grown in Man Rogosa Sharpe (MRS) and M17 (Liofilchem srl, Roseto degli Abruzzi (TE), Italy) broth, incubated at 37 °C and stored at −80 °C in the same broth media, supplemented with 20% (*w*/*v*) of glycerol. In order to determine the phases of the microbial growth, 1 mL of an overnight culture of each LAB strain (OD_600_ ≈ 0.82) was inoculated into 10 mL of MRS or M17 and incubated at 37 °C. Cell enumeration was determined immediately after inoculation and during incubation time (after 2, 4, 6, 8, 10 and 24 h). Serial dilutions were performed, plated into MRS and the plates were incubated for 24 h at 37 °C. The experiment was performed in triplicate.

### 2.2. Evaluation of Functional Traits

#### 2.2.1. Tolerance to Acidic Conditions

LAB strains were tested for tolerance at different pH values, as previously reported [29,30]. Acidic tolerance was tested as follows: overnight cultures were centrifuged, washed twice with phosphate-buffered saline (PBS) at pH 7.0, and re-suspended to reach a final density of 10^8^ bacteria/mL in MRS broth acidified, using HCl (1 M), at pH 3.0 and pH 2.0. One mL of each culture was diluted and plated on MRS and M17 agar. The viable bacterial counts were detected at initial time and after 3 h of incubation at 37 °C, and the survival rate was calculated and expressed as survival rate percentage (SR%), based on the initial and the final number of viable cells. The experiment was performed in triplicate.

#### 2.2.2. Bile Salt Tolerance

Bacterial cells from an overnight culture were collected by centrifugation and re-suspended in MRS broth added with 0.5 and 2.0% of bile salts (Oxgall, Sigma-Aldrich, L’lsle D’Abeau Chesnes, France), respectively and anaerobically incubated at 37 °C. MRS broth without bovine bile salts was used as a control. Aliquots were taken at initial time and after 4 h, diluted and plated on MRS agar. The survival rates were determined as:

SR (%) = (N1/N0) × 100

where N1 (Log CFU/mL) is the total viable count of selected strains after 4 h and N0 (Log CFU/mL) the total viable count of selected strains at the initial time. The experiment was performed in duplicate.

#### 2.2.3. Pepsin Tolerance

LAB cultures in MRS broth, incubated at 37 °C for 24 h, were centrifuged (10,000 rpm for 10 min), washed twice with PBS and re-suspended to a final concentration of 10^8^ bacteria/mL in MRS or M17 broth at pH 2.0 and pH 3.0, supplemented with 3 mg/mL of pepsin (Sigma-Aldrich, Germany). The acidification of media was performed using HCl (1 M). The broth cultures were incubated at 37 °C, and aliquots of 100 µL were sampled at the initial time and after 3 h, inoculated into MRS agar and incubated for 48 h at 37 °C. Viable cells were counted for the determination of survival rate (SR%), according to the above-mentioned equation [29]. The experiment was performed in triplicate.

#### 2.2.4. Auto-Aggregation Assay

The auto-aggregation assay was performed as previously described [30]. In detail, overnight cultures were centrifuged at 5000 rpm for 10 min at 4 °C. The pellets were washed twice with PBS 20 mM, pH 6.0 and re-suspended in the same buffer. Equal volumes of each bacterium were vortexed for 10 s and incubated at 30 °C for 24 h, without agitation. The auto-aggregation activity was calculated using the equation:

% Auto-aggregation = 1 − (A1/A0) × 100

where A1 and A0 were the absorbance of LAB cultures after and before incubation. The experiment was performed in triplicate.

### 2.3. Evaluation of Safety Traits

#### 2.3.1. DNase, Gelatinase and Hemolytic Assays

For detection of DNase, as previously reported by Mokdad and co-workers [31], strains were streaked in duplicate on DNase agar (Sigma, St. Louis, MO, USA) and plates were incubated at 37 °C for 48 h. After flooding, the plates with 1 N HCl, the positive indication of DNase production was visualized as a clear pink zone around the colonies.

The gelatinase activity was investigated using Nutrient agar medium (Liofilchem srl, Roseto degli Abruzzi (TE), Italy) containing 3.0% (*w*/*v*) of gelatin (Sigma Aldrich, Germany), as previously reported by Pino and co-workers [30]. All analyses were performed in duplicate.

Hemolytic activity was determined on Columbia agar medium (Biolife, Novi Ligure (AL), Italy), containing 5.0% of sheep blood, by streaking each overnight LAB culture on the surface of the solid medium. The plates were then incubated, under aerobic conditions, at 37 °C for 24–48 h and visually examined for the green zone (α-hemolysis), clear zones (β-positive) or no zone around colonies (γ-negative), as previously reported [32]. *Streptococcus pyogenes* ATCC 19615 and *Streptococcus pneumoniae* ATCC 6303 were used as positive controls.

#### 2.3.2. Antibiotic Susceptibility Test

For the seven LAB strains the antibiotic susceptibility was tested using a minimum inhibitory concentration (MIC) assay, as previously described [33]. The LAB strains were considered resistant or sensitive to each tested antibiotic (ampicillin, vancomycin, gentamycin, kanamycin, streptomycin, erythromycin, clindamycin, tetracycline, and chloramphenicol), according to breakpoints proposed by the European Food Safety Authority [34]. LAB strains were incubated at 37 °C for 24 h in the LSM formulation medium, constituted by 90% of IST broth (Iso-Sensi test broth, Oxoid Ltd., UK) supplemented with 10% of MRS broth. Each strain was standardized, by using a 1.0 McFarland standard solution, to reach the final inoculum density of 10^8^ CFU/mL. 100 µL of each standardized culture were used and incubated for 48 h at 37 °C, in the absence (positive control) and in the presence of each antibiotic at the following concentration (mg/L) ranges: ampicillin (1–32), vancomycin (0.5–16), gentamicin (4–128), kanamycin (8–256), streptomycin (8–256), erythromycin (0.125–4), clindamycin (0.125–4), tetracycline (1–32), and chloramphenicol (1–32). An aliquot of 100 μL of antibiotic, diluted into an LSM medium with the composition mentioned by ISO 10932/IDF 223 [33,35], was inoculated with 100 μL of bacterial inoculum in a microplate (Euroclone SpA, Pero, Italy). The MIC was determined as the lowest antibiotic concentration where no visible growth was observed [36]. All analyses were performed in duplicate.

### 2.4. Antibacterial Activity

#### 2.4.1. Target Strains and Growth Conditions

The antimicrobial activity was evaluated against a panel of five target bacteria, including *Listeria monocytogenes* ATCC 19114, *Escherichia coli* ATCC 25922, *Salmonella enterica* Typhimurium ATCC 14026, and *Pseudomonas aeruginosa* ATCC 3224 and *Staphylococcus aureus* ATCC 29213. The target strains were revitalized in Luria–Bertani (LB) medium (Liofilchem, Roseto degli Abruzzi (TE) Italy) at 37 °C.

#### 2.4.2. Antibacterial Activity of LAB Strains (as Cells or CFSs)

Antibacterial activity of LAB strains was tested, by both agar-well diffusion and dilution methods, against the same target bacteria (namely *L. monocytogenes* ATCC 19114, *E. coli* ATCC 25922, *S*. Typhimurium ATCC 14026, *P. aeruginosa* ATCC 3224, and *Staphylococcus aureus* ATCC 29213). The turbidity of the microbial suspensions was adjusted to 0.5 McFarland, giving an approximate cell density of 1.5 × 10^8^ CFU/mL [37]. The target cultures were spread on LB agar medium, using a cotton swab, and 50 µL of LAB cultures were injected on wells (6 mm diameter), previously cut into agar plates. Likewise, 50 µL of both crude supernatants, obtained by centrifugation (10,000 rpm for 20 min at 4 °C) of the LAB cultures, grown overnight at 37 °C in MRS broth, and deacidified supernatant (adjusted to pH 6.5 with 1 N of NaOH to rule out acidic inhibition), filtered through a 0.22 µm membrane filter (Millipore, Billerica, MA, USA) were injected on wells. The plates were aerobically incubated at 37 °C for 24 h and the antibacterial activity was evaluated by the measurement of the inhibition diameter around the well (≤ 5 mm, low inhibition; 5–10 mm, medium inhibition, ≥10 mm, high inhibition) [38].

The dilution method was performed using the broth microdilution assay in a 96-well plate, as previously reported [39]. 100 µL of both standardized LAB cultures (10^8^ CFU/mL) and diverse cell density of target bacteria, fixed from 10^6^ to 10^3^ CFU/mL, were inoculated in a mixed media of MRS and BHI (Brain Heart Infusion) broth (Liofilchem srl, Roseto degli Abruzzi (TE), Italy) and co-incubated for 48 h at 37 °C. After incubation, both LAB and target bacteria were enumerated on MRS and BHI agar, respectively. Single cultivation of each microorganism was used as a control. The experiments were performed in triplicate.

### 2.5. Fruit Processing and Treatments

#### 2.5.1. Vegetal Matrix

Pomegranate fruits (*Punica granatum* L. *cv* Jolly Red) harvested in autumn 2021, at the commercial ripening stage, and classified as C4 category [40] were used. The fruits were kindly provided by a local grower located in Bronte (Sicily) on the same day of harvesting. Fruits were transferred to the laboratory of Di3A (at the University of Catania) and immediately treated.

#### 2.5.2. Fruit Treatments

Arils were manually extracted with a knife and washed in water containing sodium hypochlorite solution (0.5% *w*/*v*) for 2 min. The washed arils were then collected and placed in sterile containers. Arils that presented blemishes were removed. Afterwards, arils were dried and weighed at portions of 100 g under aseptic conditions, in an ordinary atmosphere, into transparent polystyrene bags PS 6 (code: V00501/OPS) with the following characteristics L: 127 × 115 mm; 1: 45 mm; 0.08 m^3^.

To evaluate the bio-preservative effect of CFS, fruits were inoculated with a mixed culture of *L. monocytogenes* ATCC 19114 and *E. coli* ATCC 8739 (ratio 1:1 at ~10^4^ CFU/100 g) and subsequently sprayed with the CFS obtained from *L. fermentum* LBF4 (OM165034) and *L. fermentum* LBF5 (OM165035) cultures. Moreover, in order to compare the antimicrobial effect of CFS to citric acid, samples (100 g) of pomegranate arils inoculated with the mixed culture of *L. monocytogenes* ATCC 19114 and *E. coli* ATCC 8739 (ratio 1:1 at ~10^4^ CFU/100 g) were sprayed with a fresh solution of 3% (*v*/*w*) of citric acid. Samples without CFS neither citric acid were considered as controls. All samples were packaged, as described above, stored at refrigerated conditions (4 °C) for up to 12 days, and analyzed at the initial time (T0), after 3 (T3), 7 (T7), and after 12 (T12) days of storage. Physicochemical and microbiological analyses were performed at each sampling time.

#### 2.5.3. Physicochemical Analyses

The weight loss of pomegranate aril samples was detected after 3, 7 and 12 days of refrigerated storage. The weight of samples was measured using a precision electronic scale accurate to two decimal places (Gibertini EU-C 2002 RS, Novate Milanese, Italy) with an accuracy of ±0.20 g. Weight loss (WL) was calculated using the equation:

WL = (W0 − W1)/W0 × 100

where W0 is the initial weight (g) and W1 is the final weight (g).

The firmness of pomegranate aril samples was determined using a Universal machine (Zwick/Roell DO-FB0.5 TS model 2002, Genoa, Italy) and referred as the maximum force required for breaking the fruit skin. The firmness was evaluated in fresh fruits and in minimally processed samples, differently treated, at an initial time and after 3, 7 and 12 days of storage at 4 °C. The firmness analyzer was equipped with a 5.8 mm diameter cylinder probe (P6); the set parameters of each test were: pre-test speed 2 mm/s, test speed 0.5 mm/s and distance of 2 mm, post-test speed 4 mm/s and force max 1 N. For each treatment, three replicates of five arils were measured. The maximum force of the peak reached during aril tissue breakage was registered as firmness and expressed in Newton (N).

The colorimetric analyses were performed in fresh fruits and in minimally processed samples, differently treated, at an initial time and after 3, 7 and 12 days of storage at 4 °C. The color coordinates L* (brightness), a* (green-red component), and b* (blue-yellow component) were detected and reported as the average of three transmittance measurements for a single replicate, using a CM-5 spectrophotometer (Minolta, Milan, Italy), with the D65 illuminant, according to the CIELAB scale. Color was assessed according to the Commission Internationale de l’Éclairage (CIE) and expressed as L*, a*, b* color values. The coordinates L*, a* and b* indicate the lightness of the color (L* = 0 and L* = 100 represented black and white, respectively), its position between green and red (negative and positive a* values indicate greenness and redness, respectively) and between blue and yellow (negative and positive b* values point towards blueness and yellowness, respectively).

Once firmness and color were determined, the arils were blended, and the juice was stored at −20 °C for further analyses.

The pH of juice obtained from fresh fruits and from minimally processed samples, differently treated, at an initial time and after 3, 7 and 12 days of storage at 4 °C, was measured using a Mettler DL25 pH meter (Mettler-Toledo International Inc., Columbus, OH, USA).

The total soluble solids (TSS) of juice obtained from fresh fruits and from minimally processed samples, differently treated, at an initial time and after 3, 7 and 12 days of storage at 4 °C, were determined using a refractometer (Atago, RX-5000, Fisher Scientific, Rodano (MI), Italy), and expressed as °Brix.

The organic acid content of juice, obtained from fresh fruits and from minimally processed samples, differently treated, at an initial time and after 3, 7 and 12 days of storage at 4 °C, was determined by injecting the samples filtered through a 0.45 μm PTFE syringe filter (Merck, Frankfurter Str. 250, 64293 Darmstadt, Germany) into the chromatographic HPLC system.

In addition, the organic acid profile of *L. fermentum* LBF4 (OM165034) plus *L. fermentum* LBF5 (OM165035) supernatant (CFS) was also determined. The system consisted of a Waters Alliance 2695 HPLC liquid chromatograph, equipped with a Waters 996 photodiode array (PDA) detector set at 210 nm and Waters Empower software (Waters Corporation, Milford, MA, USA). Isocratic elution with 5 mM sulfuric acid was performed on a Rezex ROA Organic Acid H+ column (Phenomenex, Torrence, CA, USA) maintained in an oven at 26 °C. The run time was set at 50 min with a flow rate of 0.6 mL/min. The quantification of the peaks was obtained through calibration curves of pure standard of lactic, citric, acetic, propionic, isobutyric and butyric acids (all purchased from Sigma-Aldrich, Italy) injected at different concentrations. All analyses were performed in triplicate for each sample.

### 2.6. Microbiological Analyses

The microbiological analyses were performed according to Thakur and Sharma [32], with slight modifications. In detail, 10 g of each pomegranate sample were aseptically collected and homogenized in sterile bags (Whirl-Pak1, Nasco, Fort Atkinson, WI, USA), containing 90 mL of physiological water (0.9% NaCl), using a stomacher 80 lab blender (Seward Ltd., West Sussex, England). After performing serial decimal dilutions, a volume of 0.1 mL was plated onto appropriate microbiological media: EC X-gluc (Biolife srl, Novi Ligure (AL), Italy) for *E. coli* and Listeria Palcam Agar for *L. monocytogenes* enumeration and plates were incubated for 48–72 h at 37 °C. The same procedures were performed for control samples. Randomly, in order to confirm species affiliation, additionally basic microbiological tests, such as Gram staining, microscopic observation and catalase tests were carried out on colonies growth on selective media. In addition, total mesophilic microorganisms (TMM) and total psychrotrophic microorganisms (TPM) were counted on plate count agar (PCA) and incubated at 32 °C for 24 h and at 4 °C for 5 days, respectively. All media were purchased from Liofilchem srl (Roseto degli Abruzzi (TE), Italy). Results were expressed as a mean of log10 CFU/g ± standard deviation (SD) of two replications.

### 2.7. Statistical Analyses

All data were expressed as mean ± standard deviation (SD). Statistical significance was determined using an analysis of variance (ANOVA). The values were considered as significantly different when *p* ≤ 0.05. The SPSS software (version 21.0, IBM Statistics, Armonk, NY, USA) was used for data processing. Statistical analysis of the obtained results was performed using one-way analysis of variance (ANOVA), and Tukey’s HSD post-hoc test for means separation at a significance level of *p* ≤ 0.05. To evaluate the relationships among the different physical, chemical and microbiological parameters of minimally processed arils, differently treated, data were subjected to a one-tailed Pearson’s correlation.

## 3. Results

### 3.1. Growth Rate of LAB Strains

The tested LAB proliferated immediately after inoculation into MRS and M17 broth media. No significant differences in growth rate among LAB strains were observed; they showed a short lag phase (about 2 h) and an exponential phase that lasted up to 8 h. *L. fermentum* LBF15 showed the highest biomass yield, reaching a density around 7.4 Log CFU/mL (data not shown).

### 3.2. Functional Traits Assessment

#### Acidic, Bile Salt, Pepsin Tolerance and Auto-Aggregation

Regarding acidic tolerance, starting from an initial number of viable cells (control cells) ranging from 6.80 to 6.47 Log CFU/mL, a survival rates ≥ 90% were observed at pH 3.0. The survival rate at pH 2 was slightly lower, showing values between 85.74 and 96.67%. Significant differences were observed among LAB strains (*p* ≤ 0.01), except for the ability to survive at pH 3.0 (Table 1).

Results of bile salt tolerance, shown in Table 2, revealed that after 3 h of incubation, LAB strains highlighted significant differences (*p* ≤ 0.01), exhibiting high viability with a survival rate always above 90%, at 0.5% (*w*/*v*) of bile salt concentration, and between 87.79 and 96.82% at 1.0% (*w*/*v*) of bile salt presence. Lower survival rates were observed for the highest tested bile salt concentration (2%), except for LBF15 and LBF5 strains which showed values close to 92.89 and 89.47%, respectively (Table 2).

Data on pepsin tolerance (Table 3) revealed no significant differences (*p* > 0.05) in the ability to survive at both tested pH values. All strains showed a good resistance at both pH 3.0 and pH 2.0, when 3 mg/mL of pepsin were added, reaching a survival rate always higher than 90% (Table 3).

**Figure 1:**
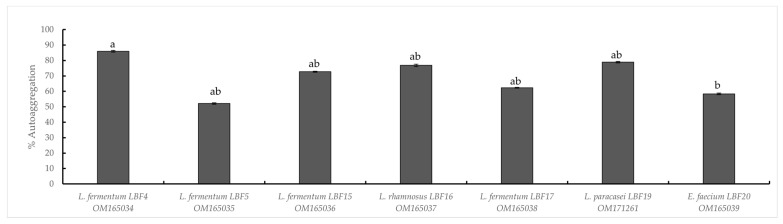
Autoaggregation of LAB strains (as a percentage). Data are expressed as means ± SD. Different letters indicate significant differences of means (*n* = 3) based on Tukey’s HSD test at *p* ≤ 0.05.

As shown in Figure 1, all tested strains exhibited auto-aggregation activity, ranging from 60% to 85%. *L. fermentum* LBF4 showed the highest auto-aggregating value, as 85% after 24 h of incubation at 30 °C (Figure 1).

### 3.3. Safety Evaluation

#### DNase, Gelatinase, Hemolytic Activity and Antibiotic Resistance

None of the tested LAB strains showed the ability to produce DNase and gelatinase, or to exert hemolytic activity, fulfilling such safety requirements. Results on antibiotic susceptibility, evaluated by MIC method, are shown in Table 4. In details, *L. fermentum* LBF4, *L. fermentum* LBF5 and *L. rhamnosus* LBF16 strains were sensitive to all tested antibiotics, while *L. paracasei* LBF19 showed resistance to streptomycin and chloramphenicol. The remaining strains, *L. fermentum* LBF15 and LBF17 were resistant to ampicillin, kanamycin and erythromycin and, as expected, *E. faecium* LBF 20 showed resistance towards ampicillin, vancomycin, streptomycin, tetracycline and chloramphenicol (Table 4).

### 3.4. Antibacterial Activity of Lab Strains

Results of antibacterial activity showed that, overall, both the cells and the CFSs obtained from *L. fermentum* LBF4, *L. fermentum* LBF5, and *L. rhamnosus* LBF16 displayed the highest inhibition against *L. monocytogenes* ATCC 19114, *E. coli* ATCC 8739, and *S.* Typhimurium ATCC 14026 (Supplementary Table S1). After all, *L. fermentum* LBF5 exhibited the highest inhibition effect also against *P. aeruginosa* ATCC 3224. Although with different alone sizes, *L. fermentum* LBF4 and *L. rhamnosus* LBF16 showed the highest inhibition effect against *S. aureus* ATCC 29213. A similar inhibitory effect against all target bacteria was observed for *E. faecium* LBF20.

No activity was detected for the neutralized CFSs against any tested target bacteria, highlighting that the antagonistic activity is attributable to the acidic content (mainly lactic, citric and acetic acids).

**Figure 2:**
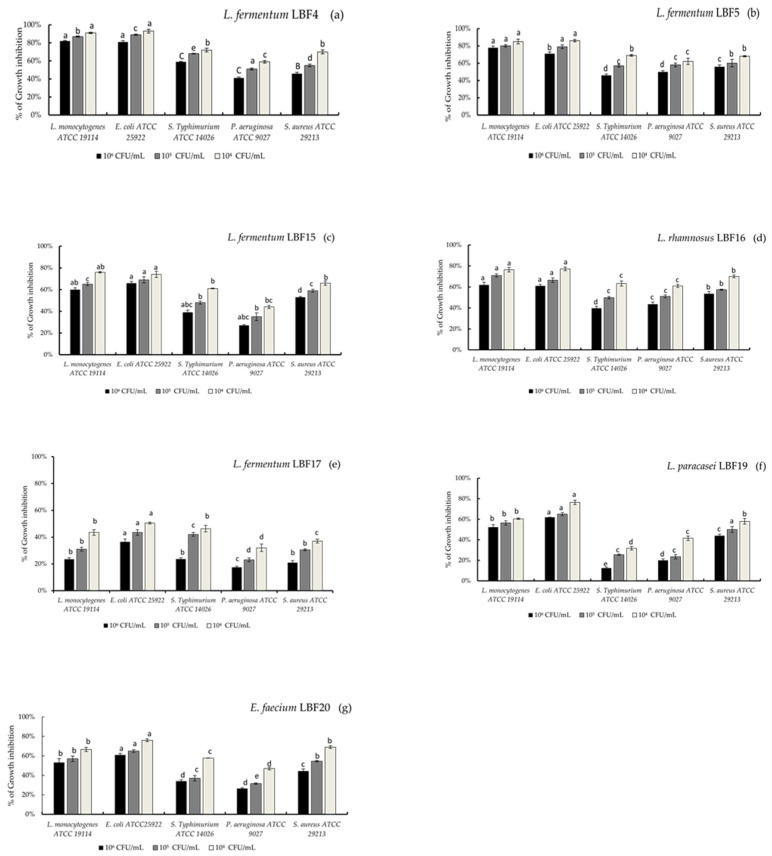
Antimicrobial activity (as percentage of growth inhibition) of *L. fermentum* species (**a**–**c**,**e**), *L. rhamnosus* species(**d**), *L. paracasei* species (**f**), *E. faecium* (**g**) species against target bacteria at different concentrations in co-culture.

Regarding the inhibition activity against the target bacteria in a liquid medium, the results were independent on the bacterial species and highlighted no influence caused by co-cultivation with the pathogenic bacteria, remaining after 24 h of incubation, always constant (Figure 2, panels from a to g). Overall, for all LAB strains a complete inhibition of target bacteria at 10^3^ CFU/mL concentration was observed (data not shown). Among the tested strains, the *L. fermentum* LBF4 and *L. fermentum* LBF5 exhibited the strongest inhibitory effect against *E. coli* ATCC 8739 and *L. monocytogenes* ATCC 19114, at both 10^5^ and 10^4^ CFU/mL concentrations, with an inhibition rate higher than 80% (Figure 2, panels a and b). Similar behaviour was observed for *L. rhamnosus* LBF16 and *L. paracasei* LBF19 strains, while for *E. faecium* LBF20, an inhibition rate slight lower against *E. coli* ATCC 8739 at 10^4^ and 10^5^ concentrations was observed (Figure 2, panel d, f, and g). Finally, *L. paracasei* LBF19 and *E. faecium* LBF20 showed an inhibition rate around 60% against *L. monocytogenes* ATCC 19114 at both tested concentrations (Figure 2, panels f and g). After all, strains showed an inhibition rate lower than 60% against the remaining tested target bacteria at any concentrations (Figure 2, panels from a to g).

### 3.5. Physicochemical Analyses of Minimally Processed Pomegranate Arils Differently Treated during Storage at Refrigerated Conditions

**Figure 3:**
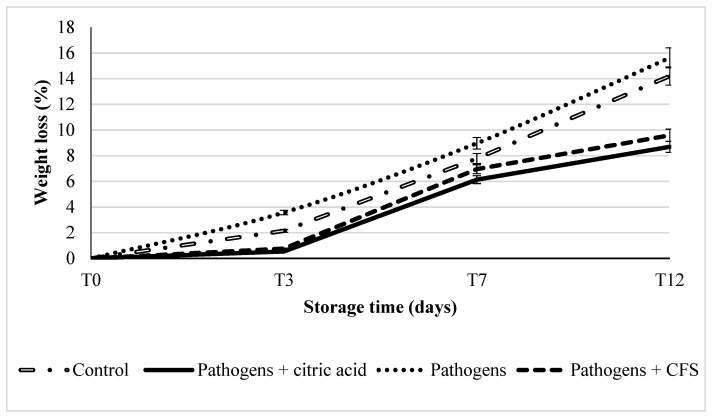
Weight loss (%) in pomegranate aril samples was differently treated during storage at refrigerated conditions.

During storage, the weight of the pomegranate arils constantly decreased (Figure 3), mainly starting from the third day. Nevertheless, the weight loss did not exceed the 1% value until the third day of storage in samples treated with CFS or citric acid. For control samples and for untreated contaminated samples, the initial weight loss exceeded 2% and 3%, respectively (Figure 3). Increasing of weight loss was detected without any significant difference (*p* > 0.05) within the untreated (inoculated and control) samples, reaching 14% and 15.6%, respectively, on the twelfth day of storage. In contrast, in treated samples (with the addition of citric acid or CFS), the weight loss reached values of 8.7% and 9.6%, respectively after 12 days of storage. Indeed, as expected, in samples inoculated with pathogens and without any treatments, the weight loss was positively related with both *E. coli* and *L. monocytogenes* cell densities (R = 0.847 and R = 0.805, respectively), whereas in samples inoculated with pathogens and treated with CFS a negative correlation (R= −0.779) was found for *L. monocytogenes*.

Regarding physicochemical traits, the fresh pomegranate fruits revealed a firmness (N) value of 10.18 ± 1.39 (Table 5), which decreased in all processed samples after 3 days of storage (Table 5), reaching final values, scored between 6.38 and 7.48, at the end of storage time, with any significant difference among samples. A different behaviour was observed after 7 days in samples without any treatments (control and samples inoculated with pathogens), which showed values of firmness of 9.15 and 9.05, respectively, higher than values detected in treated samples, with citric acid (7.72) or with CFS (7.08) (Table 5).

The color coordinates of fresh fruits and pomegranate aril differently treated, detected at the initial time and during storage, are reported in Table 5. The effects of treatments and storage time were not significant for lightness (L*) values (*p* > 0.05) after 3 and 7 days. Similarly, the analysis of variance showed that no significant effect among samples on all colorimetric coordinates, used as a color stability indicator, was observed after 7 days of storage. For all samples, the redness values increased after 7 days and decreased after 12 days of storage, resulting markedly higher than that found in fresh fruits. On the contrary, for samples inoculated with pathogens, the values increased during the whole storage period. Regarding the b* index, although the values detected in control samples decreased during the storage, both treated and contaminated samples showed an increase after 7 days of storage, followed by a decrease at the end of storage (Table 5).

Chemical data obtained from fruits, CFS and minimally processed arils, during storage time, are reported in Table 6. Focusing on CFS, results showed the presence of lactic (6788.38 mg/L), citric (3023.48 mg/L), acetic (2878.54 mg/L), and propionic (31.63 mg/L) acids. The same organic acids were detected in samples differently treated at different storage times. Moreover, the presence of butyric acid revealed only in CFS at 1476.23 mg/L, was never detected in any samples, neither in aril samples inoculated with pathogens and treated with CFS.

Overall, the chemical results showed a significant effect of treatments during the storage period (*p* ≤ 0.05). Focusing on pH, the values of all tested samples decreased during storage (Table 6), whereas no significant difference was observed for TSS value until the seventh day of storage. Indeed, on the twelfth day of storage, the TSS values decreased, reaching the lowest values in samples inoculated with pathogens (14.7 mg/L).

Regarding the acid content, the storage time showed a significant effect on citric and lactic acids for all treatments (*p* ≤ 0.05), significantly decreasing in all samples mainly at the twelfth day of storage. Conversely, no significant difference was detected for propionic acid content in any samples, both at initial and final storage times, while after 7 days of storage the lowest values were observed (Table 6). After all, the presence of acetic acid, revealed only in samples inoculated with pathogens starting from the third day of storage, increased in all samples at the seventh day of storage, showing significant differences at the twelfth day, when the highest values were reached in samples inoculated with pathogens and treated with CFS or citric acid.

### 3.6. Microbiological Analyses of Minimally Processed Pomegranate Arils Differently Treated during Storage at Refrigerated Conditions

**Figure 4:**
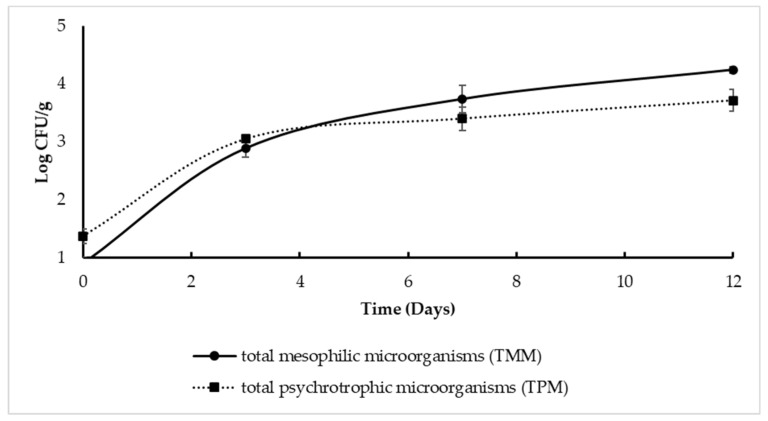
Total mesophilic microorganism (TMM) and total psychrotrophic microorganisms (TPM) densities in pomegranate aril samples without any treatment (controls) during storage at refrigerated conditions.

**Figure 5:**
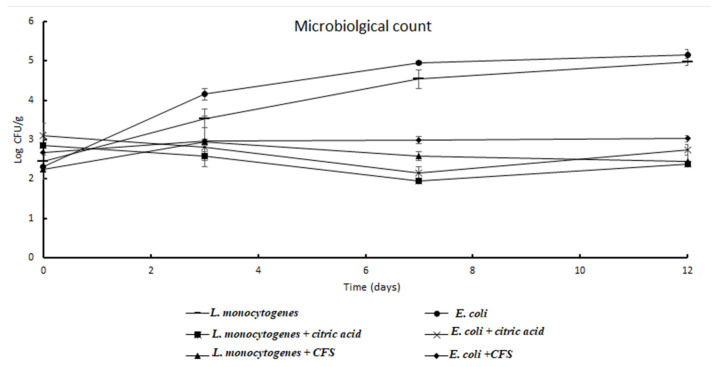
*L. monocytogenes* and *E. coli* counts in pomegranate aril samples were differently treated during storage at refrigerated conditions.

Results of microbiological analyses performed on minimally processed pomegranate arils are shown in Figure 4 and Figure 5. In control samples (Figure 4), the initial total mesophilic microorganisms (TMM) and the total psychrotrophic microorganisms (TPM) densities were found as 0.9 ± 0.1 Log CFU/g and 1.3 ± 0.12 Log CFU/g, respectively, indicating satisfactory microbiological traits. During storage, the TMM and TPM counts increased, reaching values of 4.19 and 3.73 Log CFU/g, respectively, after 12 days of refrigerated conditions.

Focusing on *L. monocytogenes*, the mean initial density in pomegranate aril samples (2.5 Log CFU/g) increased during the storage time. In particular, in control samples, presumptive *L. monocytogenes* density climbed to 3.54 Log CFU/g after 3 days, to reach the value of 4.98 Log CFU/g, after 12 days of storage, with an increase in 2.53 Log units. In samples inoculated with pathogens, the treatment with citric acid resulted in a decrease in almost 0.5 Log units with a final count, after 12 days, of 2.37 Log CFU/g, with the highest listericidal effect between the third and the seventh day of storage. Regarding the pomegranate aril samples inoculated with pathogens, the treatment with CFS resulted in a slight increase (0.72 Log unit) after 3 days, followed by a weak decrease after 7 and 12 days, when the *L. monocytogenes* density was found as 2.45 Log CFU/g. A similar trend was observed for *E. coli* counts. In samples inoculated with pathogens, without any treatment, the presumptive *E. coli* count jumped from an initial 2.3 to final 5.12 Log CFU/g, with an increase of 2.85 Log units. In samples treated with citric acid, a 0.49 Log unit reduction was detected up to 12 days of refrigerated conditions, whilst in samples treated with CFS a final value of 3.0 Log CFU/g was detected, with an overall increase in 0.33 Log units.

## 4. Discussion

Due to the change of lifestyles, a relevant increase in the request of fresh-cut horticultural produce has been observed [41,42] and the major challenge facing the fresh-cut industry is to find natural alternatives to the chemicals, in order to prevent browning and microbial growth, which still represent major health concerns and cause economic losses [43,44]. Among the various natural strategies based on the bioprotection [42], the use of post-biotics has emerged. The proposal of applying probiotic metabolites has the advantage, for consumers, to lower the risk of infection and for the food industry, to be easy to use lowering the interactions with the food matrix [20,21,22].

In the present study, seven LAB strains were proposed as potential probiotic bacteria based on the safety evaluation and their ability to survive at acidic conditions, in the presence of bile salts and pepsin [45]. The latest result is in contrast with a previous report [46], where no strain was viable after exposure to pH 2.0, when 3 mg/mL of pepsin was added.

All strains showed high autoaggregation scores (above 60%), as recently reported for lactobacilli and enterococci [39,47].

Overall, the seven strains were sensitive to all tested antibiotics with MIC lower than EFSA breakpoints, except the two *L. fermentum* strains (LBF15 and LBF17), which were slightly resistance to ampicillin, kanamycin and erythromycin and *L. paracasei* LBF19 that was resistant to streptomycin and chloramphenicol. Whereas, *E. faecium* LBF20 showed resistance to ampicillin, vancomycin, streptomycin, tetracycline and chloramphenicol, although Russo and co-workers [33] observed a high incidence of resistance to different antibiotics (namely rifampicin, erythromycin and gentamicin) within the *Enterococcus* genus.

The tested strains showed no hemolytic or DNase activity, satisfying the two most relevant safety criteria for probiotic selection [47,48,49].

In the present study, bio-protective cultures of selected LAB strains against a broad spectrum of target bacteria, using both agar-well diffusion and co-culture methods, were explored. Overall, both LAB and CFSs showed antagonistic activity against target microorganisms, although the inhibition effect varied among LAB and target strains, in agreement with previous reports [18,50,51]. The most relevant effect was observed against *E. coli* and *L. monocytogenes*. Indeed, in co-culture, the two strains (LBF4 and LBF5) showed a significant reduction of *E. coli* and *L. monocytogenes* densities, with an inhibition rate higher than 79%. Furthermore, a bactericidal effect was observed when a lower density of target bacteria was used, as previously reported [52].

Results obtained from CFS demonstrated that the inhibition effect was due to the organic acid contents, as previously reviewed by Mani-López and co-workers [53]. The in vitro efficacy of the CFSs of the most promising LAB strains was confirmed on minimal processed pomegranate arils inoculated with a mixed culture of *L. monocytogenes* and *E. coli*.

Indeed, the CFSs addition resulted in a microbial density reduction, for *E. coli* and *L. monocytogenes*, as counted CFU, comparable to the citric acid addition.

According to our results, the weight loss was highest in samples inoculated with pathogens and lowest in samples inoculated with pathogens and treated with citric acid.

Focusing on texture, the firmness detected in arils was not significantly different among samples (*p* > 0.05), mainly after 3 and 12 days of storage, indicating that it is not affected exclusively by the percentage of water loss. In fact, the slight differences found at the seventh day of storage could be related to the higher microbial metabolism of both the naturally present microbiota and inoculated pathogens. Similar results were reported when heat-shrinkable films were applied on pomegranate fruits [54,55] or in fruits differently packaged [56]. Moreover, the quality of pomegranate arils is strongly related to their red color, which mainly depends on the cultivar and on the ripeness state. In the present study, no significant effect among treated samples (*p* > 0.05) was found on colorimetric coordinates. Although the red parameter decreased in all samples, an increase in samples treated with CFS was exhibited, supposing a correlation between the CFS and a greater stabilisation of the anthocyanin component [57,58]; this finding was confirmed for the b* parameter, which showed a decrease in yellow in all samples, except in sample containing CFS.

As expected, during the whole storage a reduction in organic acid was observed, as previously reported [59], although, starting from the seventh day of storage, the acetic acid appearance was detected. Looking at Pearson’s test, a negative correlation was observed in untreated samples inoculated with pathogens, between lactic acid and *L. monocytogenes* and *E. coli*. (R= −0.787 and R= −0.814, respectively), highlighting that lactic acid could be used as a source of nourishment. Moreover, in samples inoculated with pathogens and treated with citric acid, the pathogen densities were negatively affected by citric acid content (with an R value of −0.753 and −0.783, for *L. monocytogenes* and *E. coli*, respectively) and even more by propionic acid (with an R value of −0.960 and −0.956, for *L. monocytogenes* and *E. coli*, respectively). Considering the propionic acid content detected in fresh arils and in CFS, its increased concentration revealed in minimally processed arils, starting from the third day of storage, could be related to the metabolism of anaerobic bacteria.

Focusing on pH values, a slight decrease was detected during the whole storage, explainable by the effect of refrigeration or treatments on fruit respiration rate. Moreover, results confirmed that the CFS treatment slightly reduced the pH of pomegranate aril samples after 3 days of storage, confirming that the direct addition of a post-biotic mixture avoids the synergistic activities between organic acids and other metabolites [20,21,22].

In addition, although several organic acids were found in pomegranate aril juice, citric acid is reported as the most relevant in many varietal accessions, as found by Cirillo and co-workers [60] and by Feng et al. [61], through transcriptomic analyses.

The TSS includes mainly sugars, commonly used as maturation index, that are relevant in affecting the shelf-life of fruits [60,62]. In non-climacteric fruits, like pomegranates, a reduction in TSS content is commonly reported during storage [63]. However, in the present study, the TSS content remained constant, suffering a slight decrease at the end of storage, which can be explained by the observed water loss, as revealed by the negative correlation (R = −0.988) between TSS and percentage of weight loss in control samples. However, different studies reported not significant effects of cold storage on TSS content in different minimally processed fruits [64].

## 5. Conclusions

Results of the present study showed a great variability on in vitro probiotic, safety and antibacterial activities among the tested LAB strains. For their promising probiotic features, two *L. fermentum* strains were selected and the bio-preservative effect of their CFS, used in a mixed solution, was proven, for the first time, on pomegranate arils inoculated with *L. monocytogenes* and *E. coli.* Results revealed the good vocation of the Jolly Red cultivar to be transformed into minimally processed fruits. Moreover, based on physicochemical and microbiological results, the CFS of selected strains can be proposed as a bio-preservative for pomegranate arils with a clean label.

However, the CFS stability, as well as its shelf-life, needs to be better understood and a specific legislation is required to fix the allowed limit, the method of application/addition and the CFS classification as an ingredient or additive.

## Figures and Tables

**Table 1:** Tolerance to acidic conditions.

LAB Strains	Viable Count (Log CFU/mL)				Survival Rate (%)	
	T0		T3		SR pH 2	SR pH 3
	pH 3	pH 2	pH 3	pH 2		
*L. fermentum* LBF4	6.80 ± 0.01 a	4.60 ± 0.22 c	6.48 ± 0.08	4.17 ± 0.28 d	90.65	95.29
*L. fermentum* LBF5	6.47 ± 0.03 c	6.60 ± 0.11 a	6.34 ± 0.16	6.38 ± 0.05 a	96.67	97.99
*L. fermentum* LBF15	6.80 ± 0.02 a	5.75 ± 0.05 b	6.42 ± 0.05	5.33 ± 0.03 b	92.69	94.41
*L. rhamnosus* LBF16	6.47 ± 0.03 c	5.66 ± 0.03 b	6.15 ± 0.27	5.08 ± 0.11 ab	89.75	95.05
*L. fermentum* LBF17	6.60 ± 0.07 b	5.51 ± 0.19 b	6.37 ± 0.09	5.07 ± 0.07 ab	91.01	96.51
*L. paracasei* LBF19	6.57 ± 0.01 bc	5.79 ± 0.10 b	6.25 ± 0.02	5.38 ± 0.16 b	92.91	95.13
*E. faecium* LBF20	6.52 ± 0.02 bc	5.54 ± 0.05 b	6.17 ± 0.03	4.75 ± 0.05 c	85.74	94.63
Sig.	**	**	n.s.	**		

Data are expressed as means ± SD. Different letters in the same column indicate significant differences based on Tukey’s HSD test. ** Significant at *p* ≤ 0.01; n.s. not significant.

**Table 2:** Tolerance to bile salt presence at different concentrations.

LAB Strains	Viable Count (Log CFU/mL)						Survival Rate (%)		
	T0			T3					
	0.5%	1.0%	2.0%	0.5%	1.0%	2.0%	SR 0.5%	SR 1.0%	SR 2.0%
*L. fermentum* LBF4	8.72 ± 0.03	7.43 ± 0.07 a	6.27 ± 0.11 b	8.56 ± 0.05 a	7.17 ± 0.07 a	5.52 ± 0.31 ab	98.16	96.50	88.04
*L. fermentum* LBF5	8.81 ± 0.01	6.33 ± 0.31 c	5.51 ± 0.04 c	8.61 ± 0.18 a	6.02 ± 0.10 b	4.93 ± 0.00 b	97.73	95.10	89.47
*L. fermentum* LBF15	8.93 ± 0.08	7.15 ± 0.21 ab	6.75 ± 0.05 a	8.68 ± 0.03 a	6.79 ± 0.11 a	6.27 ± 0.02 a	97.20	94.96	92.89
*L. rhamnosus* LBF16	8.52 ± 0.14	5.02 ± 0.34 d	2.79 ± 0.04 d	8.29 ± 0.28 ab	4.69 ± 0.33 c	2.38 ± 0.31 c	97.30	93.43	85.30
*L. fermentum* LBF17	8.14 ± 0.18	7.54 ± 0.00 a	6.66 ± 0.01 a	7.91 ± 0.01 b	7.30 ± 0.01 a	5.51 ± 0.04 ab	97.17	96.82	82.73
*L. paracasei* LBF19	8.61 ± 0.07	6.53 ± 0.07 bc	5.74 ± 0.16 c	8.33 ± 0.16 ab	6.21 ± 0.03 b	5.06 ± 0.64 b	96.75	95.10	88.15
*E. faecium* LBF20	8.49 ± 0.52	6.47 ± 0.12 bc	6.41 ± 0.10 ab	8.10 ± 0.11 ab	5.68 ± 0.02 b	5.25 ± 0.02 ab	95.41	87.79	81.90
Sig.	n.s.	**	**	**	**	**			

Data are expressed as means ± SD. Different letters in the same column indicate significant differences based on Tukey’s HSD test. ** Significant at *p* ≤ 0.01; n.s. not significant.

**Table 3:** Tolerance to pepsin at different pH values.

Strains	Viable Count (Log CFU/mL)				Survival Rate (%)	
	T0		T3		SR pH3	SR pH2
	pH 3	pH 2	pH 3	pH 2		
*L. fermentum* LBF4	7.22 ± 0.25 a	6.83 ± 0.05 a	6.97 ± 0.26	6.38 ± 0.22	96.54	93.41
*L. fermentum* LBF5	6.55 ± 0.01 b	6.54 ± 0.05 abc	6.39 ± 0.04	6.23 ± 0.03	97.56	95.26
*L. fermentum* LBF15	6.72 ± 0.03 b	6.79 ± 0.11 ab	6.42 ± 0.02	6.29 ± 0.00	95.54	92.64
*L. rhamnosus* LBF16	6.59 ± 0.02 b	6.43 ± 0.03 c	6.38 ± 0.16	6.14 ± 0.33	96.81	95.49
*L. fermentum* LBF17	6.56 ± 0.04 b	6.60 ± 0.08 abc	6.37 ± 0.54	6.31 ± 0.04	97.10	95.61
*L. paracasei* LBF19	6.56 ± 0.07 b	6.60 ± 0.08 abc	6.35 ± 0.53	6.32 ± 0.02	96.80	95.76
*E. faecium* LBF20	6.60 ± 0.11 b	6.47 ± 0.12 bc	6.38 ± 0.06	6.20 ± 0.09	96.67	95.83
Sig.	**	*	n.s.	n.s.		

Data are expressed as means±SD. Different letters in the same column indicate significant differences based on Tukey’s HSD test. ** Significant at *p* ≤ 0.01; * Significant at *p* ≤ 0.05; n.s. not significant.

**Table 4:** Antibiotic susceptibility of LAB strains.

Strains	Antibiotic (µg/mL)								
	AMP (≥2 ^a^)	VAN (^nr^)	GEN (≥16 ^a^)	KAN (≥32 ^a^)	STRE (≥64 ^a^)	ERY (≥1 ^a^)	CLI (≥1 ^a^)	TET (≥8 ^a^)	CHL (≥4 ^a^)
*L. fermentum* LBF4	2 ^a^	0.5 ^nr^	16 ^a^	16 ^a^	32 ^a^	1 ^a^	0.5 ^a^	4 ^a^	4 ^a^
*L. fermentum* LBF5	2 ^a^	16 ^nr^	16 ^a^	32 ^a^	64 ^a^	1 ^a^	1 ^a^	8 ^a^	4 ^a^
*L. fermentum* LBF15	4 ^R^	4 ^nr^	8 ^a^	128 ^R^	8 ^a^	2 ^R^	1 ^a^	4 ^a^	1 ^a^
*L. rhamnosus* LBF16	4 ^a^	32 ^nr^	8 ^a^	64 ^a^	8 ^a^	1 ^a^	0.5 ^a^	1 ^a^	4 ^a^
*L. fermentum* LBF17	32 ^R^	16 ^nr^	4 ^a^	256 ^R^	4 ^a^	4 ^R^	0.5 ^a^	4 ^a^	4 ^a^
*L. paracasei* LBF19	4	16 ^nr^	16 ^a^	64 ^a^	256 ^R^	0.5 ^a^	0.5 ^a^	4	16 ^R^
*E. faecium* LBF20	32 ^R^	16 ^R^	8 ^a^	256 ^a^	256 ^R^	1 ^a^	4 ^a^	16 ^R^	32 ^R^

^R^: resistant; ^a^: EFSA breakpoints; ^nr^: Not required.

**Table 5:** Texture and color coordinates of pomegranate aril samples differently treated during storage.

Samples	Time (Days)	Firmness (N)	L* (D65)	a* (D65)	b* (D65)
Fresh pomegranate arils	T0	10.18 ± 1.39	34.93 ± 1.17	5.67 ± 1.39	1.35 ± 0.57
Control	T3	7.31 ± 1.97 n.s	33.09 ± 2.19 n.s.	9.91 ± 2.44 a	3.86 ± 1.77 a
Pathogens + citric acid	T3	6.75 ± 1.94 n.s.	32.59 ± 1.09 n.s.	5.97 ± 3.58 ab	1.48 ± 1.37 ab
Pathogens	T3	7.48 ± 0.56 n.s.	32.06 ± 0.96 n.s.	6.28 ± 2.55 a	1.46 ± 0.93 ab
Pathogens + CFS	T3	6.39 ± 0.77 n.s	32.68 ± 0.94 n.s.	5.17 ± 2.31 b	1.13 ± 0.19 b
Control	T7	9.15 ± 1.56 a	34.00 ± 1.19 n.s	6.98 ± 2.24 n.s	1.54 ± 1.20 n.s.
Pathogens + citric acid	T7	7.72 ± 1.67 ab	34.22 ± 1.54 n.s.	8.91 ± 3.95 n.s	2.63 ± 1.60 n.s.
Pathogens	T7	9.05 ± 0.58 a	33.62 ± 1.60 n.s.	8.56 ± 3.95 n.s.	2.22 ± 1.45 n.s.
Pathogens + CFS	T7	7,08 ± 0.93 b	33.50 ± 1.20 n.s.	7.49 ± 1.57 n.s.	2.76 ± 1.35 n.s.
Control	T12	6.94 ± 1.99 n.s.	34.97 ± 1.73 a	6.38 ± 1.98 ab	1.62 ± 1.07 ab
Pathogens + citric acid	T12	6.75 ± 0.84 n.s.	31.73 ± 1.09 b	5.01 ± 2.03 b	0.84 ± 1.07 b
Pathogens	T12	7.48 ± 0.56 n.s.	31.64 ± 1.20 b	6.79 ± 1.37 ab	1.76 ± 0.63 ab
Pathogens + CFS	T12	6.38 ± 0.77 n.s.	32.47 ± 0.69 b	8.76 ± 2.65 a	2.00 ± 0.53 a

Data are expressed as means ± SD. Different letters in the same column for each sampling time indicate significant differences between the means (*n* = 3) based on Tukey’s test (*p* ≤ 0.05). L* (brightness), a* (green-red component), and b* (blue-yellow component). n.s. not significant.

**Table 6:** Chemical analyses and organic acids detected by HPLC in pomegranate aril samples during storage.

Sample	Time (Days)	pH	TSS (mg/L)	Citric Acid (mg/L)	Lactic Acid (mg/L)	Acetic Acid (mg/L)	Propionic Acid (mg/L)
Fresh pomegranate arils	T0	4.42 ± 0.03	19.97 ± 0.06	2443.47 ± 0.22	72.75 ± 3.45	0.00 ± 0.00	72.77 ± 0.03
CFS	T0	4.16 ± 0.05	----	3023.48 ± 30.43	6785.38 ± 50.02	2878.54 ± 67.08	31.63 ± 1.3
Control	T3	4.13 ± 0.01 a	16.94 ± 0.02 n.s	2458.12 ± 25.32 a	2155.86 ± 169.16 a	0.00 ± 0.00 b	453.05 ± 7.77 n.s.
Pathogens + citric acid	T3	3.95 ± 0.02 ab	16.31 ± 0.01 n.s	2374.97 ± 0.01 ab	1405.00 ± 33.05 b	0.00 ± 0.00 b	452.76 ± 0.89 n.s.
Pathogens	T3	3.90 ± 0.01 b	16.41 ± 0.03 n.s	2311.49 ± 41.66 b	2305.90 ± 33.12 a	228.63 ± 20.64 a	420.83 ± 23.67 n.s.
Pathogens + CFS	T3	4.12 ± 0.04 a	16.32 ± 0.02 n.s	2425.13 ± 24.64 a	1625.35 ± 92.74 ab	0.00 ± 0.00 b	372.00 ± 3.00 n.s
Control	T7	3.90 ± 0.03 a	16.31 ± 0.01 n.s	2317.06 ± 6.46 n.s.	1575.06 ± 75.89 n.s	175.50 ± 32.10 n.s.	434.90 ± 8.29 a
Pathogens + citric acid	T7	3.83 ± 0.01 b	16.06 ± 0.07 n.s	2307.32 ± 4.66 n.s	1541.71 ± 176.52 n.s	183.24 ± 8.06 n.s.	341.11 ± 9.00 c
Pathogens	T7	3.85 ± 0.01 b	16.53 ± 0.02 n.s	2314.23 ± 18.37n.s.	1767.40 ± 105.99 n.s	204.33 ± 20.23 n.s.	413.45 ± 30.75 ab
Pathogens + CFS	T7	3.86 ± 0.02 ab	16.26 ± 0.08 n.s	2386.38 ± 14.7 n.s.	1430.47 ± 203.20 n.s	159.13 ± 5.02 n.s.	380.83 ± 8.52 bc
Control	T12	3.77 ± 0.06 a	15.09 ± 0.01 b	2317.39 ± 13.45 a	1327.94 ± 201.57 a	568.72 ± 59.13 b	434.33 ± 5.51 n.s.
Pathogens + citric acid	T12	3.51 ± 0.02 c	14.94 ± 0.12 b	2288.66 ± 15.54 ab	895.26 ± 43.98 b	1072.07 ± 29.41 a	407.34 ± 16.70 n.s.
Pathogens	T12	3.66 ± 0.01 b	14.75 ± 0.08 c	2248.04 ± 12.16 b	916.13 ± 23.14 b	601.48 ± 72.15 b	368.14 ± 26.11 n.s.
Pathogens + CFS	T12	3.49 ± 0.02 c	15.33 ± 0.01 a	2287.71 ± 6.57 ab	1165.06 ± 41.77 a	1179.62 ± 8.21 a	425.32 ± 20.91 n.s.

Data are expressed as means ± SD. Different letters in the same column for each sampling time indicate significant differences between the means (*n* = 3) based on Tukey’s test (*p* ≤ 0.05). n.s not significant.

## Data Availability

All related data and methods are presented in this paper. Additional inquiries should be addressed to the corresponding author.

## Supplementary Materials

The following supporting information can be downloaded at: https://www.mdpi.com/article/10.3390/microorganisms10101876/s1, Table S1: Antibacterial activity of LAB both as cells or CFSs (expressed as mm) against target bacteria by agar-well diffusion method.
