# Supplementary material for: Multi-Functional Potential of Lactic Acid Bacteria Strains and Antimicrobial Effects in Minimally Processed Pomegranate (Punica granatum L. cv Jolly Red) Arils
Source: Microorganisms. 2022 Sep 20;10(10):1876. doi: 10.3390/microorganisms10101876 (PMC9610940; doi:10.3390/microorganisms10101876)
Supplement: Supplementary file 1 [file microorganisms-10-01876-s001.zip › microorganisms-1906373-supplementary.pdf]

Table S1. Antibacterial activity of LAB both as cells or CFSs (expressed as mm) against target bacteria by agar-well diffusion method

Strains		*Listeria monocytogenes* ATCC 19114	*Escherichia coli* ATCC 25922	*Salmonella* Typhimurium ATCC 14026	*Pseudomonas aeruginosa* ATCC 14026	*Staphylococcus aureus* ATCC 29213
L. fermentum LBF4	Cells	16.00±1.00	15.50±0.50	13.50±0.70	11.00±0.00	14.00±1.41
	CFS	20.00 0.00	20.00±1.00	15.50±0.70	12.00±1.41	16.00±1.41
L. fermentum LBF5	Cells	16.00±0.00	14.50±0.50	13.00±0.00	13.50±1.50	12.50±0.50
	CFS	19.33±0.57	18.00 ±1.00	14.66±0.57	14.50±0.70	15.33±0.47
L. fermentum LBF15	Cells	14.00±1.00	14.00±1.00	14.50±1.50	10.50±0.70	10.00±1.41
	CFS	18.00±0.00	17.00±1.00	13.00±0.00	11.00±1.00	15.50±0.50
L. rhamnosus LBF16	Cells	15.50±0.70	13.50±0.50	11.00±1.00	10.00±0.00	13.00±1.00
	CFS	18.00±0.00	17.00±1.00	14.16±1.04	14.00±0.00	16.00±0.00
L. fermentum LBF17	Cells	13.00±0.50	12.00±1.00	11.00±1.00	09.00±1.00	09.50±0.50
	CFS	12.66±057	12.00 ±1.00	11.66±0.57	10.00±0.00	10.66±0.57
L. paracasei LBF19	Cells	14.50±1.50	14.00±0.00	9.50±0.50	10.50±0.70	12.50±0.70
	CFS	17.50±0.50	17.00 ±1.00	10.5±0.70	11.50±0.70	14.25±1.06
E. faecium LBF20	Cells	15.50±1.50	15.50±0.50	11.00±1.00	10.00±0.50	14.50±0.70
	CFS	17.00±0.00	17.33 ±0.57	13.00±0.00	10.00±0.70	16.00±0.00

Data are expressed as mean ± standard deviation of three replicates.
